# Long Term Time-Lapse Imaging of Geographic Atrophy: A Pilot Study

**DOI:** 10.3389/fmed.2022.868163

**Published:** 2022-06-22

**Authors:** Michel Paques, Nathaniel Norberg, Céline Chaumette, Florian Sennlaub, Ethan Rossi, Ysé Borella, Kate Grieve

**Affiliations:** ^1^Paris Eye Imaging Group, Clinical Investigation Center 1423, Quinze-Vingts Hospital, INSERM-DHOS, Sorbonne Université, INSERM, Paris, France; ^2^Institut de la Vision, Paris, France; ^3^Department of Ophthalmology, The University of Pittsburgh School of Medicine, Pittsburgh, PA, United States

**Keywords:** age-related macular degeneration, geographic atrophy, scanning laser ophthalmoscopy, optical coherence tomography, time-lapse imaging

## Abstract

Geographic atrophy (GA), the late stage of age-related macular degeneration, is a major cause of visual disability whose pathophysiology remains largely unknown. Modern fundus imaging and histology revealed the complexity of the cellular changes that accompanies atrophy. Documenting the activity of the disease in the margins of atrophy, where the transition from health to disease occurs, would contribute to a better understanding of the progression of GA. Time-lapse imaging facilitates the identification of structural continuities in changing environments. In this retrospective pilot study, we documented the long-term changes in atrophy margins by time-lapse imaging of infrared scanning laser ophthalmoscopy (SLO) and optical coherence tomography (OCT) images in 6 cases of GA covering a mean period of 32.8 months (range, 18–72). The mean interval between imaging sessions was 2.4 months (range, 1.4–3.8). By viewing time-lapse sequences we observed extensive changes in the pattern of marginal hyperreflective spots, which associated fragmentation, increase and/or disappearance. Over the entire span of the follow-up, the most striking changes were those affecting hyperreflective spots closest to margins of atrophy, on the non-atrophic side of the retina; a continuum between the successive positions of some of the hyperreflective spots was detected, both by SLO and OCT. This continuum in their successive positions resulted in a subjective impression of a centrifugal motion of hyperreflective spots ahead of atrophy progression. Such mobilization of hyperreflective spots was detected up to several hundred microns away from atrophic borders. Such process is likely to reflect the inflammatory and degenerative process underlying GA progression and hence deserves further investigations. These results highlight the interest of multimodal time-lapse imaging to document cell-scale dynamics during progression of GA.

**Clinical Trial Registration:**

clinicaltrials.gov, identifier: NCT04128150 and NCT04129021.

## Introduction

In western countries, dry age-related macular degeneration (AMD) is a major cause of visual disability (1). In its late stage, it progresses to a stage named geographic atrophy (GA), which causes expanding zones of atrophy of the retinal pigment epithelium (RPE). The inexorable expansion of atrophic lesions may have a severe impact on quality of life, in particular when the central zone of the retina, the fovea, is involved. The risk of developing AMD results from an interplay of age, genetic background and chronic inflammation (2–5). The cellular mechanisms underlying the propagation of RPE and photoreceptor atrophy is comparatively less known.

In the margins of GA, where the transition from health to disease occurs, among the most conspicuous changes areas are hypereflective spots, commonly termed pigment mottling. In the early stages of AMD, that is, even before the occurrence of GA, pigment mottling is a biomarker of the risk of progression from early to late AMD (6–10). By histology, several phenotypes and locations of pigment mottling have been described (11–15), which can be intraretinal (therefore called hyperreflective foci, HRF), subretinal or in the subepithelial space. Along margins of atrophy, duplication of the RPE layer has also been described (16), which may account for short wavelength hyperautofluorescence seen clinically (17). Whether this pigment mottling corresponds to detached RPE cells (14, 15) transdifferentiated RPE cells (18, 19) or macrophages that have phagocytozed RPE cells (20, 21) is still a matter of debate.

The mechanisms of atrophy expansion are difficult to ascertain by histology because the latter provides only snapshots; therefore it is likely that the *in vivo* dynamics are of interest to further understand the mechanisms of progression. Time-lapse imaging, which consists of viewing a series of images registered using predefined landmarks, is a practical tool to identify structural continuities in changing environments. We previously reported that time-lapse adaptive optics ophthalmoscopy is helpful to detect motion of pigment spots during GA (22). Using time-lapse OCT, migration of pigment foci (hyperreflective foci, HRF) within the retina has also been reported (23). Here, we investigate if time-lapse sequences of SLO images could improve the characterization of the dynamic changes in fundus features associated with GA progression.

## Patients and Methods

This institutional retrospective study was carried out according to the principles outlined in the Declaration of Helsinki and was approved by an ethics committee (Comité de Protection des Personnes Sud-Est III), independent from our institution, as required by French law. The present study is an ancillary study on GA imaging registered in clinicaltrials.gov (NCT04128150 and NCT04129021). All participants gave informed consent to take part in this study. Standard procedures were used for multimodal imaging including color fundus photographs (Topcon TRC-501X) and infrared (IR) and short wavelength autofluorescence (swAF) scanning laser ophthalmoscopy (SLO), and optical coherence tomography (OCT) (Spectralis^®^, Heidelberg Engineering, Heidelberg, Germany). Near infrared autofluorescence (NIRAF) was captured using the Heidelberg Retina Angiograph (Heidelberg Engineering).

Cases were selected based on the quality of images, frequency of follow-up and presence of hyperreflective spots along atrophy margins by IR SLO. Thus, 6 eyes from 6 patients (4 females and 2 males; age range 64 to 80 years; visual acuity, counting fingers to 20/20; refraction range, −2/+0.5) fulfilling these criteria were identified. One (case 4) was reported in a previous paper (22). The mean follow-up was 32.8 months (range, 18–72); mean interval between imaging sessions was 2.4 months (range, 1.4–3.8).

SLO and OCT images were exported in portable network graphic (PNG) format. Careful registration and equalization of successive images of time-lapse videosequences is crucial to neutralize wobbling and hence document a continuum of microscopic features over time. Adjustment of brightness and contrast of each SLO frames was performed manually to smooth differences between frames using Adobe Photoshop 7.0 (Adobe Corporation, Mountain View, CA). To build up time-lapse SLO sequences, successive images were aligned with i2k Align Retina (DualAlign, LLC, Clifton Park, NY) using default settings (rigid registration). The results were evaluated by three of the authors (MP, NN, KG). Accuracy of registration and quality of equalization was based on stability of anatomical landmarks such as retinal and choroidal vessels, and the absence of scintillation. SLO scans that were obviously distorted, overexposed or out of focus were discarded. Low quality frames were easier to identify while viewing the sequence rather than separately. Residual shakes in videosequences were manually corrected using either iterative registration procedures or Photoshop. If not satisfactory, additional registration and equalization procedures were done; this process was iteratively performed as needed. Recently, we used the registration plugin of Fiji (available in the public domain at rsb.inf.nih.gov/ij; National Institutes of Health, Bethesda, MD) which improved the procedure. Then, by manually scrolling the time-lapse sequence back and forth repeatedly at various frame rates (typically 5 - 10fps), microscopic features were visually tracked individually.

Time-lapse OCT B-scan sequences were constructed in a similar way, using the Bruch's membrane and the pattern of choroidal vessels as landmarks. Building time-lapse sequences of OCT was more difficult because of the narrow plane of OCT scans and of the limitations of the performances of eye tracking of the Spectralis system; this often resulted in micrometric displacement of the plane of the scan and hence loss of features to be tracked. Other difficulties were related to the variability of the signal-to-noise ratio and to the scan deformations.

## Results

**Figure 1:**
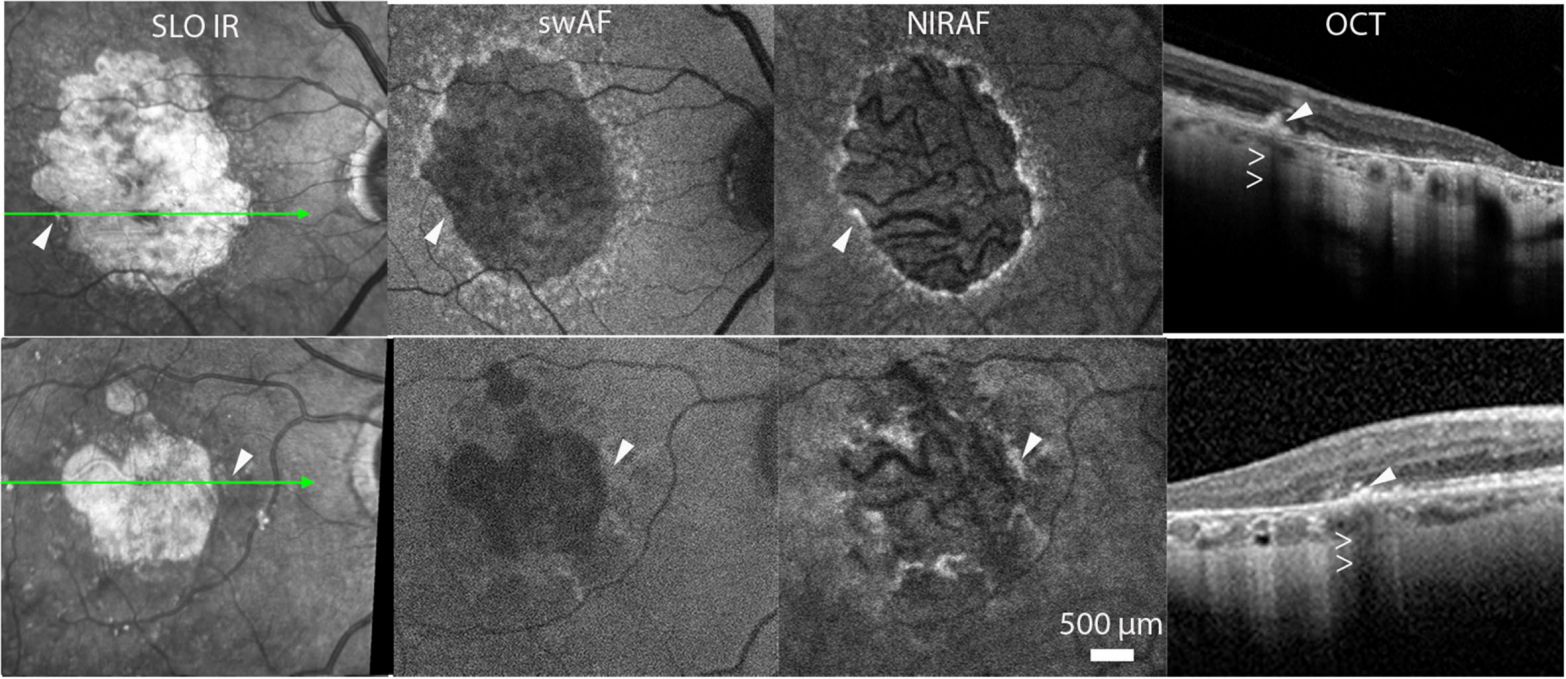
Cases 2 (top row) and 5. Multimodal imaging of GA. Arrowheads show examples of hyperreflective spots. Hollow arrowheads in OCT images point to the shadow effect.

On initial examination, the size of atrophic lesions ranged from 1.03 to 3.2 mm^2^ (average, 2.14 mm^2^). On OCT scans, atrophic margins presented features typical of complete RPE and outer retinal atrophy (24) bordered by an external limiting membrane descent. On SLO IR images a variable amount of hyperreflective spots were present along the margins of atrophy (Figure 1; Supplementary Figure 1). These spots, which varied in shape, size and amount were mostly NIRAF positive and produced a shadow effect on OCT scans. Short wavelength hyperautofluorescence was less consistent than NIRAF. Accordingly, NIRAF is believed to be both more specific and more sensitive than swAF for the detection of melanin (25–28).

**Figure 2:**
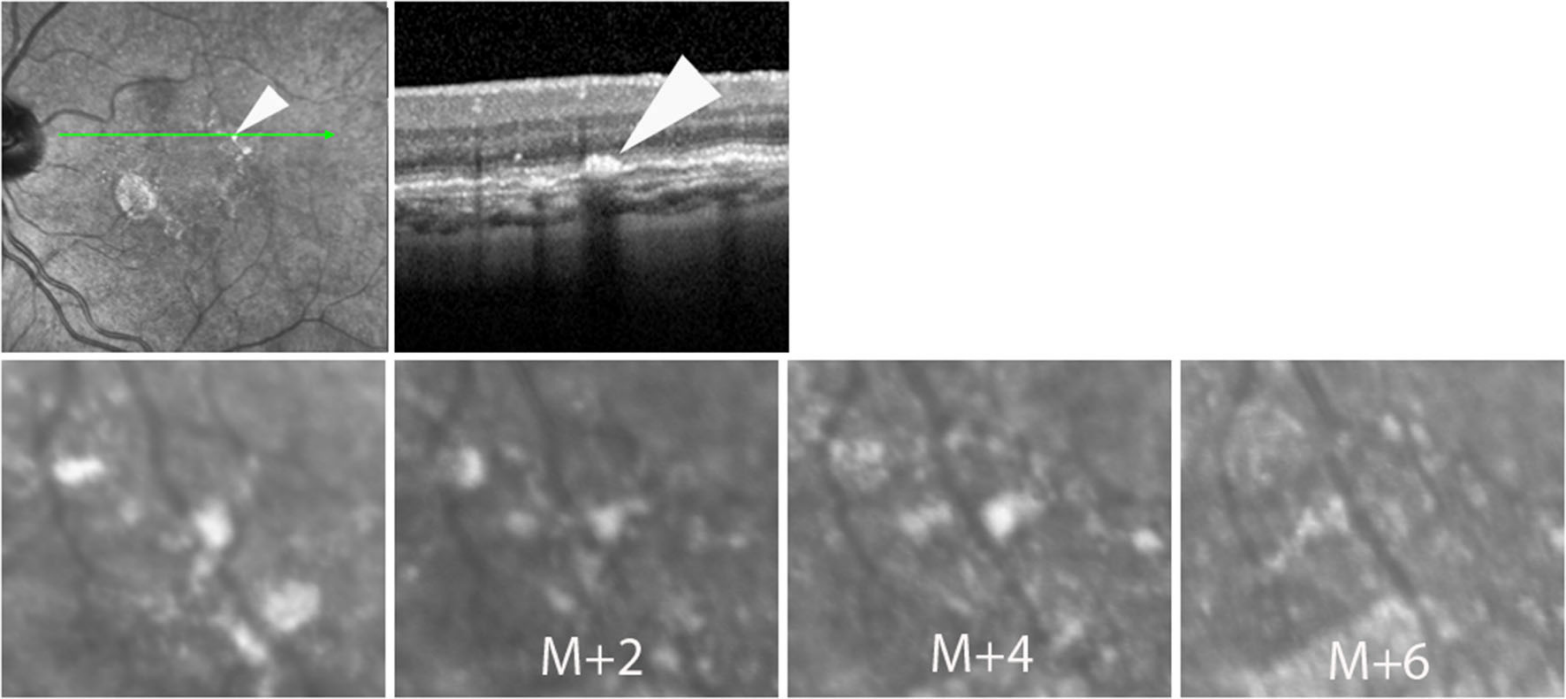
Case 4. Successive SLO images illustrating the change in the shape of a hyperreflective spot during follow-up.

**Figure 3:**
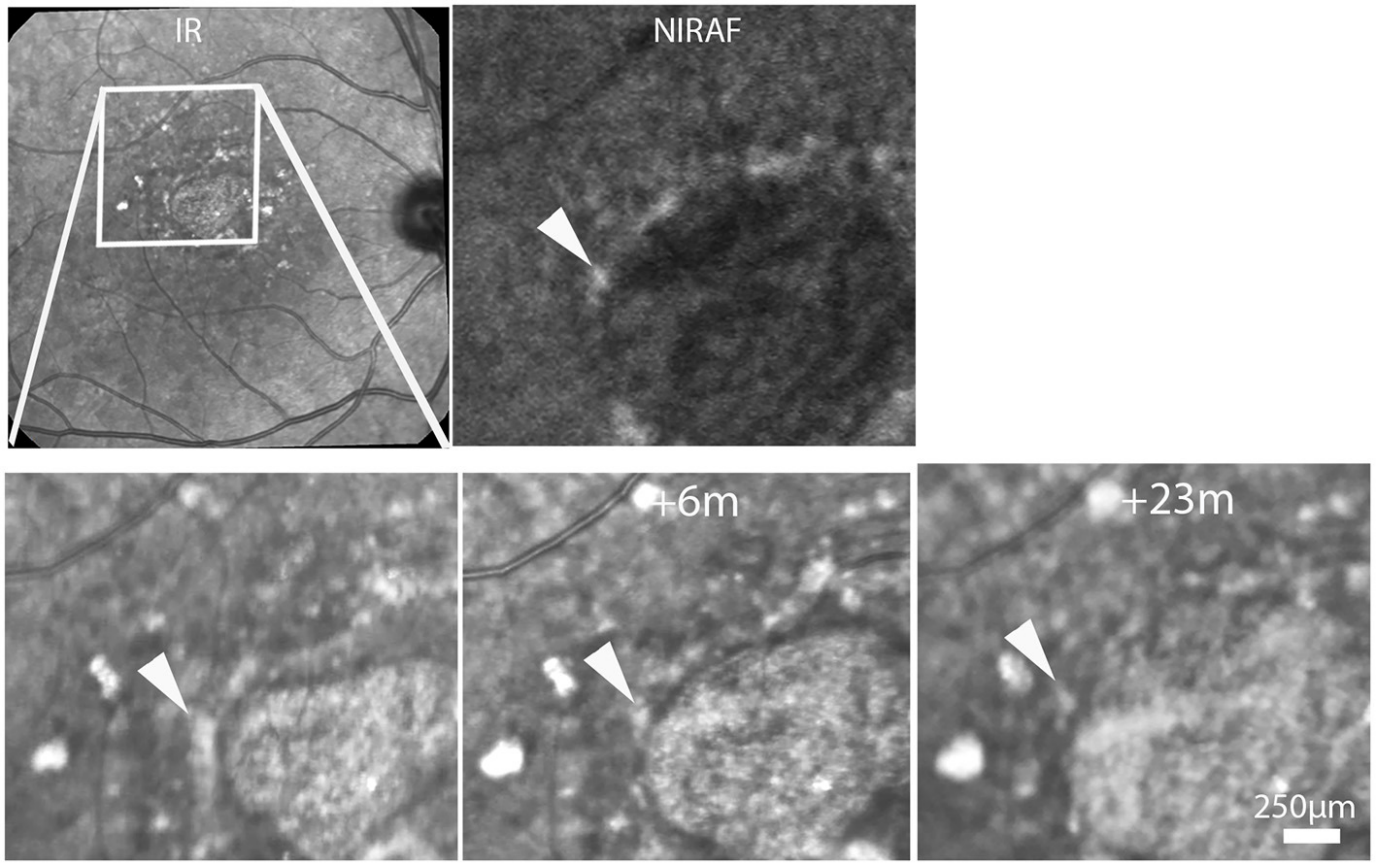
Case 6. Top row, SLO IR and NIRAF images. Bottom row, progression of atrophy. Time-points relative to the first image are indicated in the second and third images. Note the changing aspect of the hyperreflective spot (arrowheads; see also Supplementary Video 2).

During follow-up, atrophy expanded from an average surface area of 2.14 to 7.47 mm^2^ (+349 %; mean progression 1.95 mm^2^/year). The median (±SD) radial growth rate of atrophy was 127.2 μm/year (±8.7). On SLO time-lapse videosequences, atrophic areas were seen to expand in all directions (Supplementary Videos 1–3). In margins, hyperreflective spots showed extensive changes, consisting of a various association of change in shape, fragmentation, expansion or disappearance (Figure 2). Despite such variability, careful observation of the entire SLO time-lapse sequence of some of the hyperreflective spots revealed a continuum of the successive positions of hyperreflective spots (Figure 3). This continuum gave the impression of a centrifugal motion of some of these spots away from the expanding atrophic area, which is best viewed by the videos.

**Figure 4:**
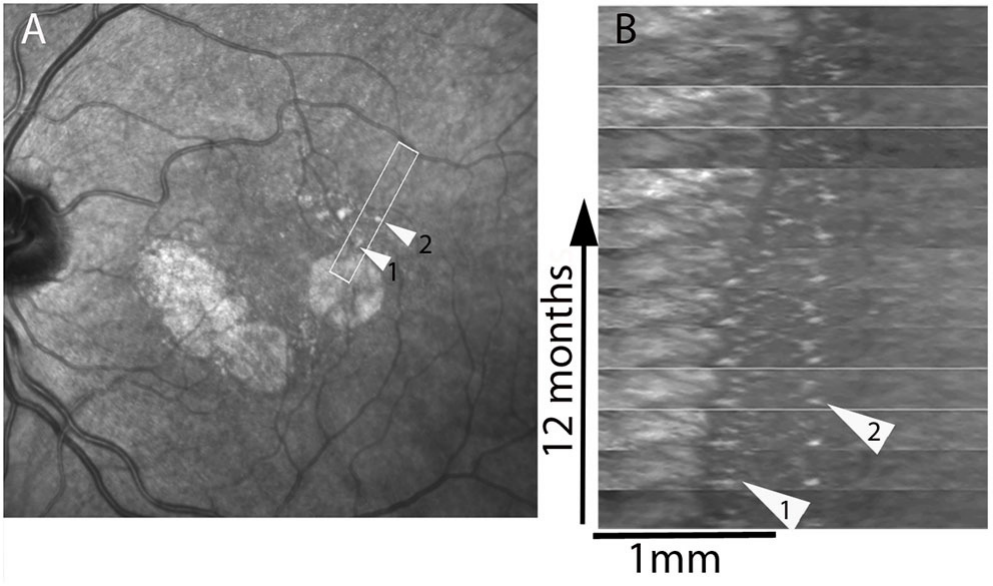
Case 4. Spatiotemporal (xt) plot. Images from regions of interest **(**boxed in **A)** were registered and rotated in **(B)** in order to display the progression from left to right; the x axis represents distance, the y axis time. Arrowheads show two hyperreflective spots that are followed-up. Note the deviation from verticality of the virtual line joining the successive positions of the hyperreflective spot shown by arrowhead 2, which is initially located 620 μm away from the margins. See also Supplementary Video 3.

To provide a more detailed view of these changes, SLO time-lapse sequences were cropped to isolate individual spots and compare their successive positions (Figure 4; Supplementary Figure 2, Supplementary Video 3). This also showed that the successive positions of these spots were temporally and spatially correlated with atrophy progression. Over the entire follow-up, that is, over several years, some spots could be tracked over distances up to from 41 to 489 μm (examples in Figure 4). In addition, some spots located more distally (i.e., several hundred microns from atrophy margins) underwent mobilization and apparent displacement away from atrophy (example in Figure 4, shown by arrow 2, Supplementary Video 3).

**Figure 5:**
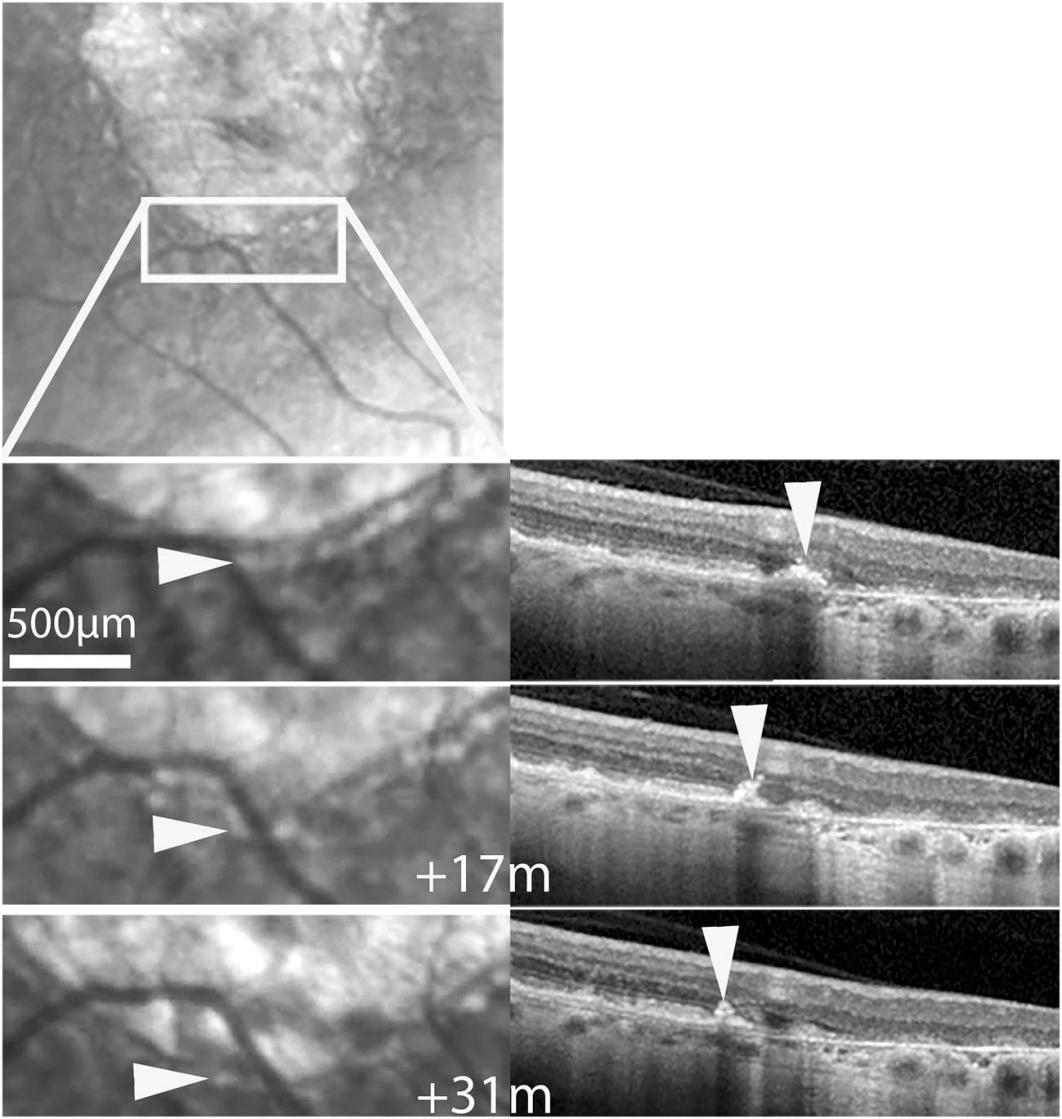
Case 2. Follow-up by SLO and OCT of a subretinalhyperreflective spot. Top, SLO IR image showing the area displayed below. Bottom shows magnifications at three time-points. Arrowheads follow a hyperreflective spot seen by SLO IR (left column) and OCT (See also Supplementary Video 5).

Time-lapse sequences of OCT scans are shown in Figure 5 and Supplementary Videos 4–6. Since OCT scans were seldom placed along the successive position of spots, we experienced more difficulties in tracking individual hyperreflective spots than by SLO. The case showed in Supplementary Video 4 shows progressive thinning of the outer nuclear layer following atrophy. Supplementary Video 4 also shows a HRF, that is, a hyperreflective spot located within the outer plexiform layer, which remained at the same location during follow-up. Two time-lapse sequences captured a subretinal hyperreflective spot (Figure 5; Supplementary Videos 5, 6). In both cases a subretinal hyperreflective spot could be detected in margins during atrophy progression. No clear evidence of upward migration (i.e., toward inner layers) was noted.

## Discussion

Here we used time-lapse sequences of SLO and OCT images to track the changes in microscopic features of margins during GA progression. We paid particular attention to the changes over time in the distribution and aspect of hyperreflective spots, a prominent feature of AMD. We observed that these hyperreflective spots are most often NIRAF positive, suggesting that they contain melanin. They were located within the RPE/Bruch's membrane complex, hence they were not what is called HRFs, which are within the retina, close to the outer plexiform layer. We observed that over the course of months these spots show conspicuous changes, either changing shape, fragmenting, growing or disappearing. Intriguingly, time-lapse imaging also revealed in many case a continuum between the patterns of hyperreflective spots. In fact, over the entire follow-up viewing the time-lapse sequence gave the impression that some of these spots underwent centrifugal displacement in synchrony with atrophy progression. This contrasted with our observation of a HRF which remained static during follow-up. In the literature, migration of a HRF toward the inner retina was previously reported (23) as well as stability over several years (19) but not centrifugal migration. Hence, taken collectively, these data suggests that subretinal hyperreflective spots and HRFs may behave differentially.

The fact that hyperreflective spots show mobilization is rather unsurprising since such motion of intraretinal cells has already been shown *in vivo* (29); it may be related to the fact that macrophages, which are migrating in response to inflammatory stimuli, are present in eyes affected by GA and may contain melanin from phagocytized RPE cells (2). The significance of the apparent continuum in the successive positions of these spots is uncertain. This does not necessarily mean that there is physical motion of these spots. Indeed, pigment deposition along margins of atrophy may be agonal changes affecting RPE cells and transmitted *neighbor to neighbor*, hence the apparent displacement could be due to propagation of RPE cell death, more or less like the propagation of a fire; however, the apparent mobilization of more distally located spots challenges this interpretation since it occurs in areas that only transformed into atrophy months later. An alternative hypothesis for centrifugal migration of subretinal hyperreflective spots could be that pigment mottling actually undergoes displacement.

Our study shows that careful construction of time-lapse sequences may be of interest to reveal the microscopic dynamic changes associated with progression of retinal diseases. Time-lapse image sequences can be constructed using commercially available software. Yet, despite the fact that some OCT systems provide built-in registration procedures in our experience there is still a need for manual processing and expert supervision, and often iterative procedures of alignment, sometime with different software to obtain satisfactory time-lapse sequences. Careful pixel-to-pixel registration and expert examination of the successive frames is indeed crucial to neutralize wobbling and hence document a continuum of microscopic features in the long term. The precision of registration is commensurate to the likeliness of distinguishing small changes from background noise. In order to track microscopic features, it is also important, to acquire images at close enough intervals. Indeed, the possibility to detect the continuity of a given feature from one time point to the next is strongly related to the time sampling, that is, the interval between two examinations. This is particularly crucial when addressing a complex and changing environment. A potential limitation of time-lapse imaging in cases with inappropriate time sampling is that it may cause false recognitions of motion patterns, similarly to a stroboscopic effect. Hence, the shorter the time between two images, the more accurate is the information brought by time-lapse imaging. It is not easy, however, to determine a priori the adequate interval to study a given process, which depends on its particular dynamics. Therefore, the interval between imaging sessions we used here for hyperreflective spots surrounding GA may not be necessarily appropriate for other disease processes. We also observed that display conditions (frame rate and back and forth) affects the possibility to detect features; in particular, back-and-forth viewing greatly improved the recognition of mobilization.

Whatever the interpretation of our data, our observations demonstrate that time-lapse sequences may be useful to investigate the long-term progression of AMD. Our findings may provide new insights into the cellular dynamics accompanying GA. Further work using time-lapse imaging may contribute to better characterize structural changes associated with GA progression. Higher resolution imaging systems such as adaptive optics ophthalmoscopy may provide a better access to the dynamics of microscopic features such as photoreceptors or RPE cells.

## Data Availability Statement

The raw data supporting the conclusions of this article will be made available by the authors, without undue reservation.

## Ethics Statement

The studies involving human participants were reviewed and approved by Comité de Protection des Personnes Région Sud-Est III (France). The patients/participants provided their written informed consent to participate in this study.

## Author Contributions

MP proposed the hypothesis, acquired data, and wrote the first draft of the manuscript. NN managed image analysis. CC acquired images. FS and ER discussed the results. KG discussed the results and wrote the manuscript. All authors contributed to the article and approved the submitted version.

## Funding

This study was funded by the Region Ile-de-France (EX047007 - SESAME 2019), the LabEx LifeSenses (ANR-10-LABX-65), the Institut Hospitalo-Universitaire ForeSIGHT (ANR-18-IAHU-01) and the Edward N. & Della L. Thome Memorial Foundation. The funding organizations had no role in the design or conduct of this research.

## Conflict of Interest

The authors declare that the research was conducted in the absence of any commercial or financial relationships that could be construed as a potential conflict of interest.

## Publisher's Note

All claims expressed in this article are solely those of the authors and do not necessarily represent those of their affiliated organizations, or those of the publisher, the editors and the reviewers. Any product that may be evaluated in this article, or claim that may be made by its manufacturer, is not guaranteed or endorsed by the publisher.

## Supplementary Material

Supplementary Figure 1 Multimodal imaging and evolution of case 1.Top row, SLO, OCT and NIRAF images showing a subretinal hyperpigmented spot (arrows). Bottom row, progression of atrophy. Time-points relative to the first image are indicated in the second and third images (see also Supplementary Video 1).

Supplementary Figure 2 Multimodal imaging and spatiotemporal (xt) plot of cases 3. Registered images from regions of interest (boxed in **A**) were extracted in order to display the progression from left to right **(B)**. Arrow points to a hyperreflective spot.

Supplementary Video 1 Time-lapse infrared SLO of case 1. Note the hyperreflective spot moving ahead of the progression front (11 frames; real time duration, 24 months). The sequence is displayed back and forth to facilitate the identification of moving features.

Supplementary Video 2 Time-lapse infrared SLO of case 6. Note the successive positions of a hyperpigmented spot (arrowhead in OCT images) moving ahead of the progression front (15 frames; real time duration, 28 months). The corresponding time-lapse OCT is shown in Supplementary Video 4.

Supplementary Video 3 Time-lapse SLO of case 4, shown in Figure 4 (16 frames; real time duration, 4 years). The boxed area is displayed in the kymograph.

Supplementary Video 4 Time-lapse OCT of case 6 (15 frames; real time duration, 28 months). Note the progressive thinning of the retina overlying the RPE loss (center of the OCT image) and the expansion of atrophy (tracked by arrows). Note also on the right a HRF in the outer plexiform layer that remains static over the entire sequence.

Supplementary Video 5 Time-lapse infrared SLO and OCT of case 2. Note the hyperreflective spot (arrowhead in OCT images) moving ahead of the progression front (9 frames; real time duration, 5 years).

Supplementary Video 6 Time-lapse infrared SLO and OCT of case 5. Note the hyperreflective spot (arrowhead in SLO and OCT images) moving ahead of the progression front (8 frames; real time duration, 18 months).
