# Supplementary figures and images for: Long Term Time-Lapse Imaging of Geographic Atrophy: A Pilot Study
Source: Front Med (Lausanne). 2022 Jun 22;9:868163. doi: 10.3389/fmed.2022.868163 (PMC9257004; doi:10.3389/fmed.2022.868163)

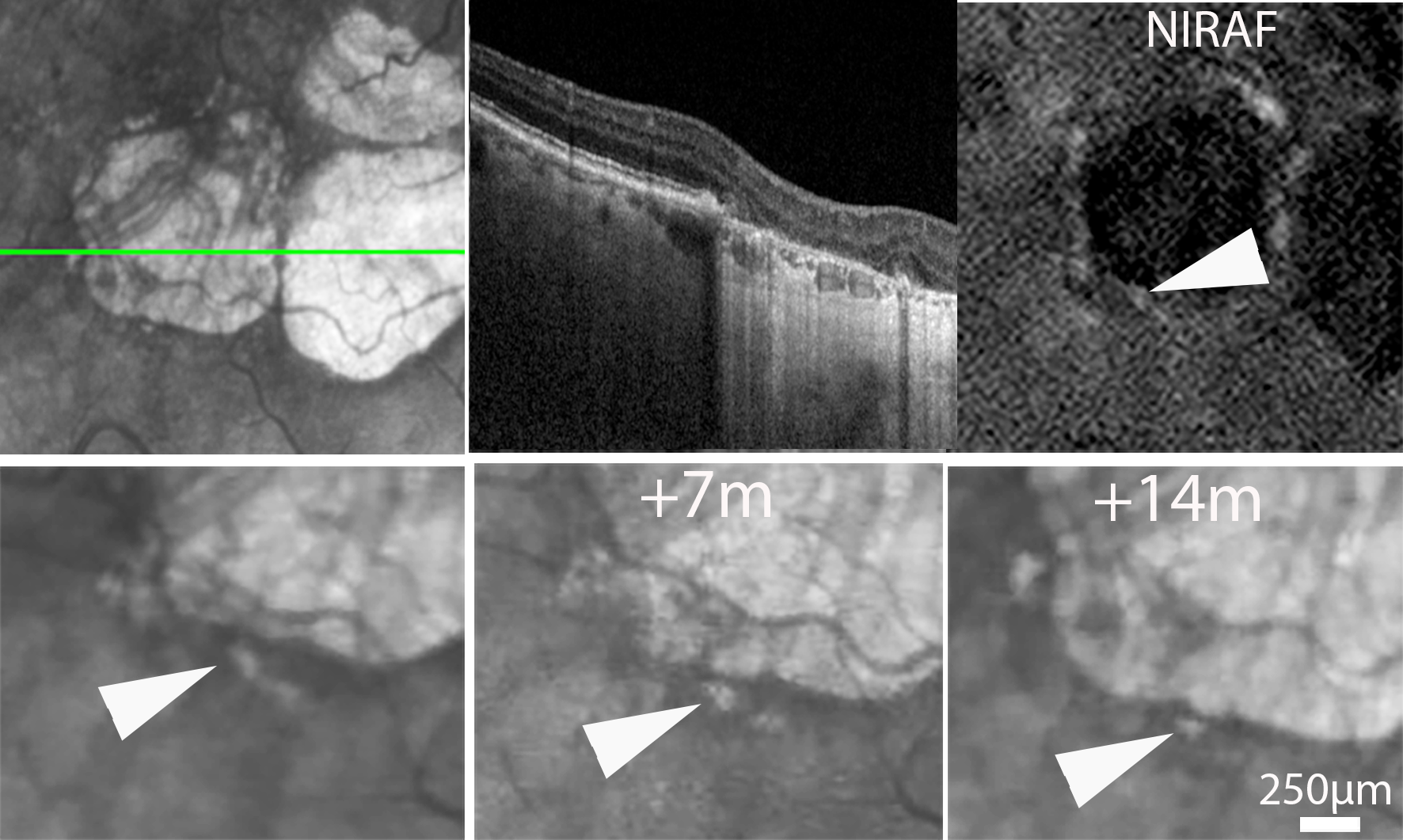

Supplement: Supplementary Figure 1 — Multimodal imaging and evolution of case 1.Top row, SLO, OCT and NIRAF images showing a subretinal hyperpigmented spot (arrows). Bottom row, progression of atrophy. Time-points relative to the first image are indicated in the second and third images (see also Supplementary Video 1). [file Image_1.tif]

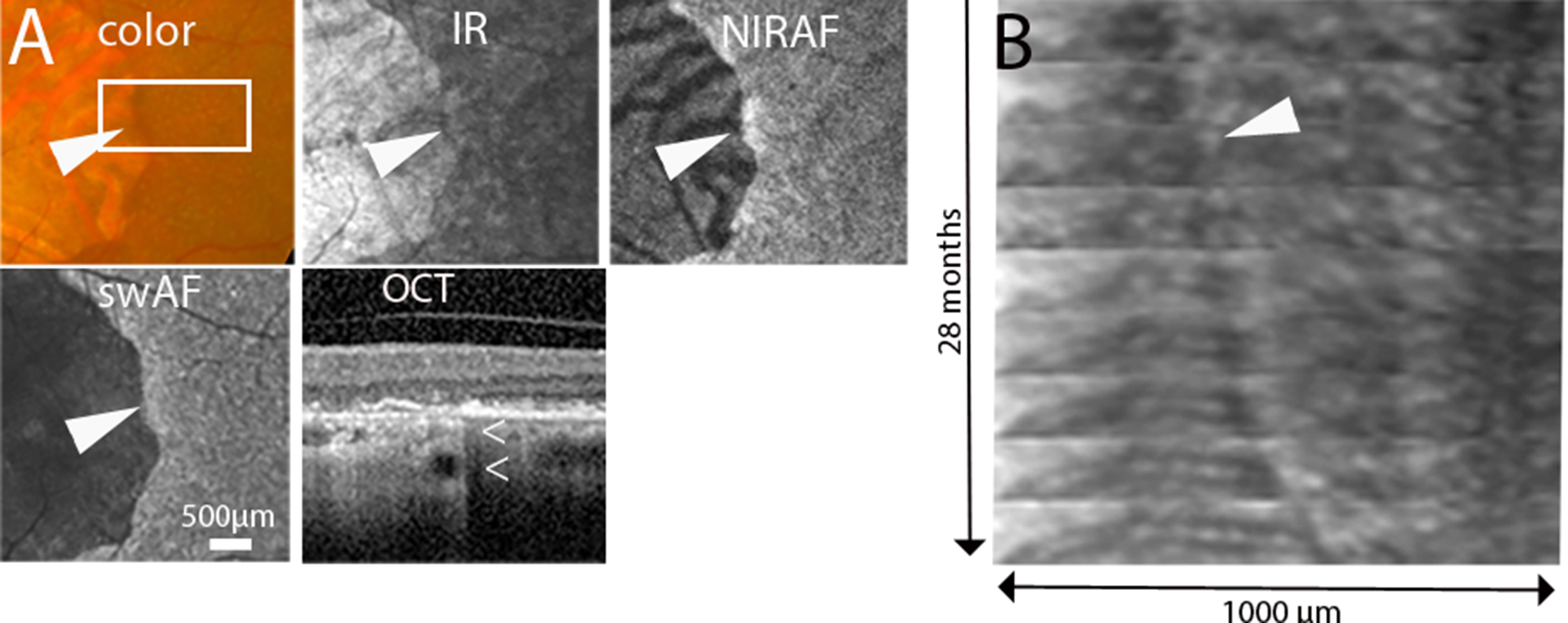

Supplement: Supplementary Figure 2 — Multimodal imaging and spatiotemporal (xt) plot of cases 3. Registered images from regions of interest (boxed in A) were extracted in order to display the progression from left to right (B). Arrow points to a hyperreflective spot. [file Image_2.tif]
